# Combination of sodium-glucose cotransporter type 2 inhibitors and urate-lowering therapy in people with gout from a specialist clinic: a retrospective, single-center observational study

**DOI:** 10.3389/fmed.2025.1726111

**Published:** 2026-01-12

**Authors:** Jose Doménech-Serrano, Ivana García-Loiseau, Cristina Rodríguez-Alvear, Pablo Riesgo-Sanchis, Óscar Moreno-Pérez, Mariano Andrés

**Affiliations:** 1 Department of Rheumatology, Dr. Balmis General University Hospital, Alicante Institute for Health and Biomedical Research (ISABIAL), Alicante, Spain; 2 Faculty of Medicine, Miguel Hernandez University, Elche, Spain; 3 Rheumatology Department, Virgen de la Peña General Hospital, Fuerteventura, Spain; 4 Departments of Endocrinology and Rheumatology, Dr. Balmis General University Hospital, Alicante, Spain; 5 Department of Clinical Medicine, University Miguel Hernandez of Elche; Alicante Institute for Health and Biomedical Research (ISABIAL), Alicante, Spain

**Keywords:** chronic kidney disease, comorbidities, diabetes mellitus, diuretics, gout, heart failure, serum urate level, sodium-glucose cotransporter type 2 inhibitors

## Abstract

To evaluate the effectiveness of sodium-glucose cotransporter type 2 (SGLT2) inhibitors combined with traditional urate-lowering therapy (ULT) in people with gout, we conducted a retrospective, single-center observational study in a crystal arthritis clinic. Primary outcomes were reduction in serum urate (SU) levels and achievement of SU targets (<6 mg/dL and <5 mg/dL). We also compared prescribed allopurinol doses versus Easy-Allo-predicted doses and evaluated changes in diuretic use. The sample comprised 46 individuals (median age 75 years; 82.6% men) with a high prevalence of long-standing gout and comorbidities. Combined treatment was associated with a median SU reduction of 0.85 mg/dL (95% CI −1.7 to −0.1, *p* = 0.001). This effect was more pronounced in individuals without prior ULT. Under combined treatment, 97.7% of participants achieved a SU level below 6 mg/dL, while 79.5% achieved a level below 5 mg/dL. Rates of target achievement did not differ by sex, age, body mass index, comorbidities, type of SGLT2 inhibitor, or initial treatment sequence. We observed a trend towards a lower prescribed versus predicted allopurinol dose and a significant reduction in diuretic use (60.9% vs. 47.8%, *p* < 0.001) after initiation of combined treatment. Combining SGLT2 inhibitors and ULT (mainly allopurinol) in people with gout achieved significant SU reductions and helped achieve treatment goals. These results, alongside lower diuretic use and possibly lower allopurinol dose requirements, point towards an interesting management approach for gout.

## 1 Introduction

Gout is the most common inflammatory arthritis in adults, with an estimated prevalence of 2.4% in Spain (1). Notably, gout impairs patients’ quality of life due to intense pain and functional limitations during flare-ups, leading to work absenteeism and high healthcare resource utilization. Furthermore, gout is closely associated with cardiovascular and renal comorbidities and increased mortality risk (2). For these reasons, the disease represents a significant public health concern.

Gout involves the deposition of monosodium urate (MSU) crystals in the joints and periarticular tissues as a result of hyperuricemia, defined as serum urate (SU) levels above 6.8 mg/dL (3). Gout is considered curable, as MSU crystal deposition is reversible when SU levels are kept below 6 mg/dL (4), with faster crystal dissolution at lower levels (5). However, real-world treatment outcomes remain suboptimal, with approximately one-third of patients in specialized care remaining above target levels (6), and poorer figures observed in primary care (7) and among patients with high vascular risk (8), where up to two-thirds of gout sufferers are not on urate target or even receive no urate-lowering agents. In addition, the mortality gap between people with gout and the general population remains unchanged over the last two decades (9). Current real-world figures contrast with clinical trial outcomes, that show >80% success in attaining urate goals (10). The main contributors include low adherence, ancestry, insufficient dose titration, and therapeutic complexity and polypharmacy in the presence of comorbidities (11, 12). Using xanthine oxidase inhibitors (XOIs), such as allopurinol or febuxostat, may be found difficult in this scenario. In chronic kidney disease (CKD), rule-based dose adjustments for allopurinol have been advocated to minimize the risk of hypersensitivity, but this has led to decreased efficacy (13). The controversy about the cardiovascular profile of febuxostat prevented its use in patients with high cardiovascular risk (14, 15). Moreover, monotherapy may be insufficient in some cases to reach the urate target in patients with high baseline urate or multiple comorbidities (for instance, fatty liver with high enzyme levels hampering dose titration). Therefore, there is a recognized gap in the treatment of patients with gout and multimorbidity, with a need for effective and safer approaches to achieving serum urate targets and improving clinical outcomes in complex populations.

Sodium-glucose cotransporter type 2 (SGLT2) inhibitors have proven benefits in people with type 2 diabetes mellitus (T2D), CKD, and heart failure, reducing morbidity and mortality, improving glycemic control, promoting weight loss, enhancing insulin sensitivity, and reducing metabolic inflammation (16). SGLT2 is expressed in the proximal renal tubule and is responsible for approximately 90% of glucose reabsorption. By blocking glucose reabsorption, SGLT2 inhibitors promote urinary glucose and sodium excretion, lowering blood glucose levels and exerting a mild diuretic and antihypertensive effect (17). Beyond glycemic control, several studies indicate a uricosuric effect. A meta-analysis of 43 randomized controlled trials showed that SGLT2 inhibitors may reduce SU levels by 33 μmol/L (0.56 mg/dL) on average (18). Mechanistically, urinary urate excretion is fostered through competition with glucose at tubular transporters, particularly GLUT9b and URAT1 (19–21). Improvement in insulin resistance and metabolic inflammation (common features of T2D, cardiovascular diseases and metabolic syndrome) may also contribute to SU reduction (22, 23). People with gout have been excluded or poorly represented in SGLT2 inhibitors trials (<5%). However, emerging observational data suggest that using SGLT2 inhibitors in individuals with T2D and gout may reduce the number of gout flares, the need for gout medication, and the number of emergency visits and hospitalizations (24, 25). Thus, the potential role of SGLT2 inhibitors in gout management deserves further investigation (26).

To date, there are no published data showing the effect of SGLT2 inhibitors combined with licensed ULT, particularly xanthine oxidase inhibitors (XOIs). By acting through two different mechanisms, this combination may enhance SU reduction and help more patients, including those with challenging comorbidities such as CKD, reach their SU targets (27). Given the lack of evidence on this treatment strategy from experimental trials, real-world data are needed to evaluate treatment outcomes in people with gout. This study aimed to evaluate the effectiveness of SGLT2 inhibitors combined with ULT in people with gout receiving care in clinical practice. Specifically, we aimed to evaluate SU reduction and the achievement of urate targets, as well as to describe the influence of covariates on treatment outcomes and assess potential allopurinol dose saving with the addition of SGLT2 inhibitors.

## 2 Methods

We designed an observational, retrospective single-center study. Participants were selected from a specialized clinic for crystal-related arthritis at a tertiary academic hospital. The study period spanned from the commercialization of SGLT2 inhibitors in Spain (2014) to December 2024. The study was approved by the Alicante-Hospital General Research Ethics Committee (ref. 2024-0109) and the Miguel Hernandez University of Elche Responsible Research Office (ref. TFG. GMEMNAC. IGL.241204). Because this was a retrospective study, we obtained a waiver to forgo informed consent from participants.

### 2.1 Eligibility criteria

The inclusion criteria were: (a) gout diagnosis, confirmed by visualization of MSU crystals under polarized microscope or by a combination of clinical and ultrasound features; (b) use of ULT with a treat-to-crystal dissolution intention; and (c) use of SGLT2 inhibitors for at least 2 months simultaneously with ULT, regardless of the indication. The drugs could be started either sequentially or simultaneously. We excluded people with no SU levels recorded 6 months before or after the initiation of combined therapy and people who received renal replacement therapy (hemodialysis, peritoneal dialysis, or transplant) during the study period.

### 2.2 Study variables

Our primary outcome variables were change in SU levels (in mg/dL) during combined therapy (continuous outcome) and achievement of SU goals set out in the 2016 European Alliance of Associations for Rheumatology (EULAR) recommendations (4): below 6 mg/dL for the average gout patient and below 5 mg/dL for people with more severe forms of gout, such as tophaceous gout, severe joint damage, or frequent flares (dichotomous outcome). SU levels were collected over the 6 months before and after combination therapy; within this period, the first SU measurement was analyzed.

Our secondary outcome variables were reduction or discontinuation of diuretics during combination therapy, the allopurinol dose (mg/day) used in clinical practice, and the theoretical allopurinol dose predicted by the Easy-Allo tool (28). This tool considers concurrent factors such as kidney function (creatinine clearance using the Cockcroft-Gault equation), body weight, baseline urate, and ethnicity (Pacific Peoples or others). We also assessed changes in other laboratory variables from 6 months before to 6 months after the initiation of combined therapy, including estimated glomerular filtration rate (eGFR; mL/min/1.73 m^2^) according to the Chronic Kidney Disease Epidemiology Collaboration (CKD-EPI) formula, serum C-reactive protein (CRP; mg/dL), fasting serum glucose (mg/dL), serum glycated hemoglobin (HbA1c; %), urine glucose (mg/dL), and urine albumin-to-creatinine ratio (UACR, mg/g).

The main explanatory variable was the combination of SGLT2 inhibitors and ULT. We collected the starting date, type, and dose (mg/day) of both agents. Other variables of interest were sex; age at treatment initiation; variables related to gout: tophaceous disease, years since first flare, use of low-dose colchicine; comorbidities: CKD according to KDIGO 2024 guidelines [eGFR <60 mL/min/1.73 m^2^ or UACR >30 mg/g (29)], hypertension, T2D, dyslipidemia, ischemic heart disease, and heart failure; smoking history; body mass index (BMI; kg/m^2^); obesity (BMI ≥30 kg/m^2^); diuretics that increase uric acid levels (loop diuretics or thiazides) with their doses; and low-dose aspirin.

We collected variables retrospectively from patients’ electronic medical records. No sample size estimation was performed given the retrospective nature of the study and the absence of previous similar works. Accordingly, the study included all patients who met our eligibility criteria.

### 2.3 Statistical analysis

For categorical variables, we first reported absolute and relative frequencies. Because the continuous variables did not follow a normal distribution according to the Kolmogorov–Smirnov test, we reported medians with their interquartile ranges (IQRs). The 95% confidence intervals (CIs) were calculated for the primary outcome variables (change in SU and achievement of SU targets).

To study differences across secondary variables according to treatment outcomes, we used the Mann–Whitney U or Kruskal–Wallis test for the non-normally distributed continuous variables, and the Chi-squared or Fisher’s exact test for categorical variables. Additionally, we explored potential baseline predictors of achieving the stricter SU target (<5 mg/dL) using a logistic regression model, which allowed us to estimate odds ratios (ORs) with their 95% CIs. Covariates with a *p* value below 0.10 in the bivariate analysis were included in the multivariable model.

We used the Wilcoxon signed-rank test to compare SU levels and other laboratory variables before and after combination treatment. We used the Mann–Whitney U test to compare the required dose of allopurinol (mg/day) versus the Easy-Allo-predicted dose (mg/day).

Analyses were performed using the SPSS Statistics software v25 (IBM, Armonk, NY). *p* values below 0.05 were considered statistically significant.

## 3 Results

Our study included 46 participants (see Table 1), mostly middle-aged to older men. There was a high prevalence of comorbidities such as T2D, CKD, and heart failure, consistent with the indication for SGLT2 inhibitors. Regarding gout characteristics, all participants had long-standing disease, and one-third had tophaceous gout.

**Table 1:** Baseline characteristics of study participants (*n* = 46) at the time of SGLT2 inhibitor and ULT combination.

Variable	*n* (%)^a^
Age in years, median (IQR)	75.0 (81%, 65.75)
Men	38 (82.6%)
Comorbidities	
Arterial hypertension	41 (89.1%)
Dyslipidemia	37 (80.4%)
Type 2 diabetes mellitus	41 (91.1%)
Chronic kidney disease	30 (65.2%)
Heart failure	21 (45.7%)
Ischemic cardiopathy	12 (26.1%)
Body mass index in kg/m^2^, median (IQR)	30.45 (33.4, 28.1)
Tobacco	14 (31.8%)
Diuretics use^b^	28 (60.9%)
Acetylsalicylic acid use	23 (50%)
Gout characteristics	
Tophaceous disease	15 (32.6%)
Years since first flare, median (IQR)	10 (26.5, 6.5)
Use of low-dose colchicine	32 (69.7%)

a Unless otherwise indicated.

b Includes loop agents and/or thiazides.

IQR, interquartile range.

ULT was initiated before SGLT2 inhibitors in 76.1% of participants (*n* = 35) and SGLT2 inhibitors before ULT in 17.4% (*n* = 8). Two participants were started on both agents simultaneously with no intermediate SU level assessment. The prescribed ULT was allopurinol in 66.7% of participants (*n* = 30) at a median dose of 300 mg/day (IQR 300, 450), febuxostat in 28.9% of participants (*n* = 13) at a median dose of 40 mg/day (IQR 40, 80), and benzbromarone in two participants (4.4%), who both received 50 mg/day. Regarding SGLT2 inhibitors, 58.7% of participants (*n* = 27) received dapagliflozin 10 mg/day, 30.4% (*n* = 14) empagliflozin (10 mg/day in eight participants and 25 mg/day in six participants), and 10.9% (*n* = 5) canagliflozin 100 mg/day.

### 3.1 Serum urate levels and achievement of serum urate targets

**Figure 1:**
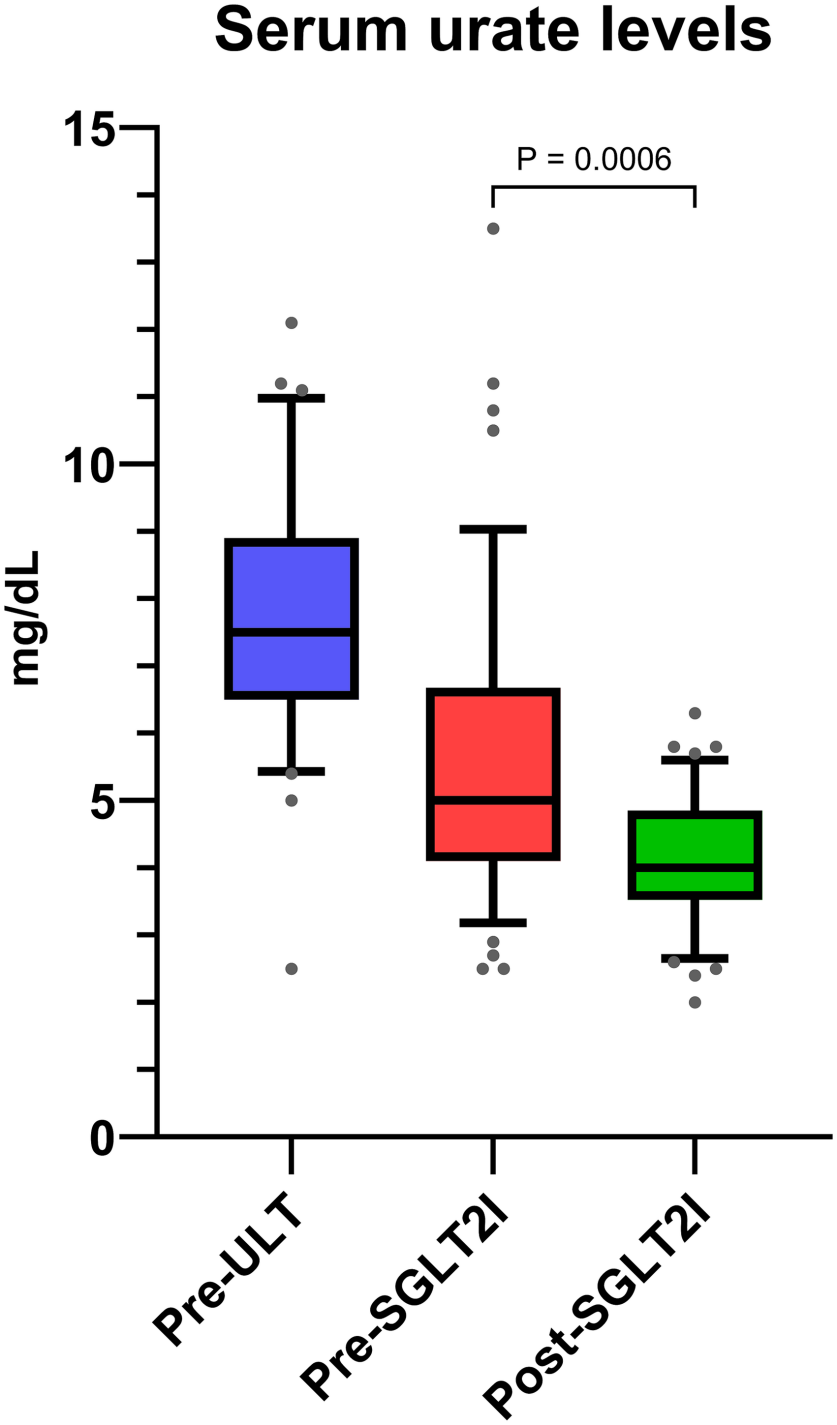
Serum urate levels. Box and whiskers plot, depicting median serum urate values (middle line in the box) with their 25th and 75th percentiles (box limits), while whiskers depict 10th and 90th percentiles. Extreme values are drawn as individual points below and above the whiskers. Data are shown for each time point: before urate-lowering therapy (Pre-ULT), before SGLT2 inhibitor initiation (Pre-SGLT2I), and after combined therapy with SGLT2 inhibitor initiation (Post-SGLT2I).

Before treatment combination, median SU levels were 5.0 mg/dL (IQR 4.1–6.8). Baseline levels were higher when the first agent prescribed was an SGLT2 inhibitor (7.2 mg/dL, IQR 5.7–11.1) than when it was a ULT agent (4.7 mg/dL, IQR 4.1–5.8). Figure 1 presents the pre- and post-combination treatment values. The use of SGLT2 inhibitors combined with ULT was associated with a median SU change of −0.85 mg/dL (IQR − 2.57, 0.45; 95% CI −1.7 to −0.1, *p* = 0.001). The reduction was significantly more pronounced in participants without versus with prior ULT, although both groups experienced a decrease, and it was observed regardless of age, sex, CKD, obesity, or type of XOI (Table 2). A weaker SU reduction was apparent with canagliflozin than the other SGLT2 inhibitors.

**Table 2:** Influence of covariates on serum urate (SU) changes and achievement of SU levels below 5 mg/dL.

Covariates	Change in SU level, Mdn (IQR) in mg/dL	*p*	SU ≥5 mg/dL, *n* (%)	SU <5 mg/dL, *n* (%)	*p*
Prior ULT					
Yes	−0.80 (−2.35 to 0.55)	**0.024**	6 (18.8%)	26 (81.3%)	1.000
No	−3.00 (−7.30 to −0.35)		1 (12.5%)	7 (87.5%)	
Diuretic adjustment^a^					
Yes	−1.30 (−2.10 to 0.20)	0.545	0 (0%)	6 (100%)	1.000
No	−1.80 (−3.90 to 0.10)		5 (21.1%)	17 (78.9%)	
Age					
<75 years	−0.95 (−2.43 to 0.35)	0.888	5 (22.7%)	17 (77.3%)	1.000
≥75 years	−0.60 (−3.13 to 0.55)		4 (18.2%)	18 (81.8%)	
Sex					
Men	−0.90 (−2.55 to 0.40)	0.851	7 (18.4%)	31 (81.6%)	0.619
Women	−0.80 (−3.90 to 0.70)		2 (25%)	6 (75%)	
BMI ≥30 kg/m^2^					
Yes	−1.0 (−2.65 to 0.55)	0.825	7 (28%)	18 (72%)	0.263
No	−0.85 (−2.85 to 0.15)		2 (11.1%)	16 (88.9%)	
CKD					
Yes	−0.90 (−2.78 to 0.40)	0.325	6 (24%)	21 (76%)	0.710
No	−0.85 (−2.18 to 0.45)		3 (15.8%)	16 (84.2%)	
Type of XOI					
Allopurinol	−0.40 (−2.55 to 0.55)	0.152	6 (20.7%)	23 (79.3%)	0.651
Febuxostat	−1.65 (−3.58 to −0.80)		1 (8.3%)	11 (91.7%)	
Type of SGLT2I					
Dapagliflozin	−1.0 (−3.35 to 0.00)	0.389	5 (20%)	20 (80%)	0.994
Empagliflozin^b^	−0.9 (−2.30 to 0.95)		2 (14.3%)	11 (78.6%)	
Canagliflozin	0.0 (−1.55 to 0.35)		1 (20%)	4 (80%)	

a Only in participants with baseline use of diuretics (*n* = 28).

b One participant on empagliflozin did not achieve the SU target.

BMI, body mass index; CKD, chronic kidney disease IQR, interquartile range; Mdn, median; SGLT2I, sodium-glucose cotransporter type 2 inhibitor; XOI, xanthine oxidase inhibitor.

Bold values indicate statistically significant association (*p* < 0.050).

With combined therapy, 97.7% of patients (95% CI 88.2 to 99.6%) achieved the standard SU target (<6 mg/dL); only one person did not meet this criterion, with an SU level of 6.3 mg/dL at the time of assessment. Notably, 79.5% of participants (95% CI 65.5 to 88.9%) also achieved SU levels below 5 mg/dL. Before initiating combined treatment, 69.6% of participants receiving ULT monotherapy had SU levels below 6 mg/dL, and 47.8% had SU levels below 5 mg/dL. In those receiving SGLT2 inhibitor monotherapy before combined treatment, only 10.9% had SU levels below 6 mg/dL, and 2.2% had SU levels below 5 mg/dL. Six months after starting combined treatment, 28.1% more participants achieved an SU level below 6 mg/dL and 31.7% more participants achieved an SU level below 5 mg/dL.

For the SU target below 5 mg/dL, no differences were detected by sex, age, obesity or CKD (Table 2). The achievement of either SU target (<6 mg/dL or <5 mg/dL) was unrelated to the first treatment prescribed (ULT or SGLT2I) or the type of XOI or SGLT2 inhibitor. However, since nearly the entire population achieved the SU levels below 6 mg/dL, potential differences between groups or factors associated with response could not be reliably evaluated.

After initiating combined treatment, the proportion of people using diuretics dropped significantly from 60.9% (n = 28) to 47.8% (*n* = 22) (*p* < 0.001). Diuretic adjustment showed no impact on SU reduction (*p* = 0.545) or the proportion of people achieving SU levels below 5 mg/dL (*p* = 1.000) (Table 2).

Regarding the predictors of achieving SU levels below 5 mg/dL, only age (OR 1.066, 95% CI 0.993–1.144; *p* = 0.079) and BMI (OR 0.858; 95% CI 0.724–1.018; *p* = 0.080) showed a trend towards a significant association with the endpoint when considered as continuous variables in the bivariate model. No other covariates showed an association (data not shown). Age and BMI were then included in the multivariate model, yielding similar results (age: adjusted OR 1.071, 95% CI 0.994–1.154, *p* = 0.072; BMI: adjusted OR 0.838, 95% CI 0.689–1.019, *p* = 0.076).

### 3.2 Allopurinol dosing and changes in other laboratory variables

**Figure 2:**
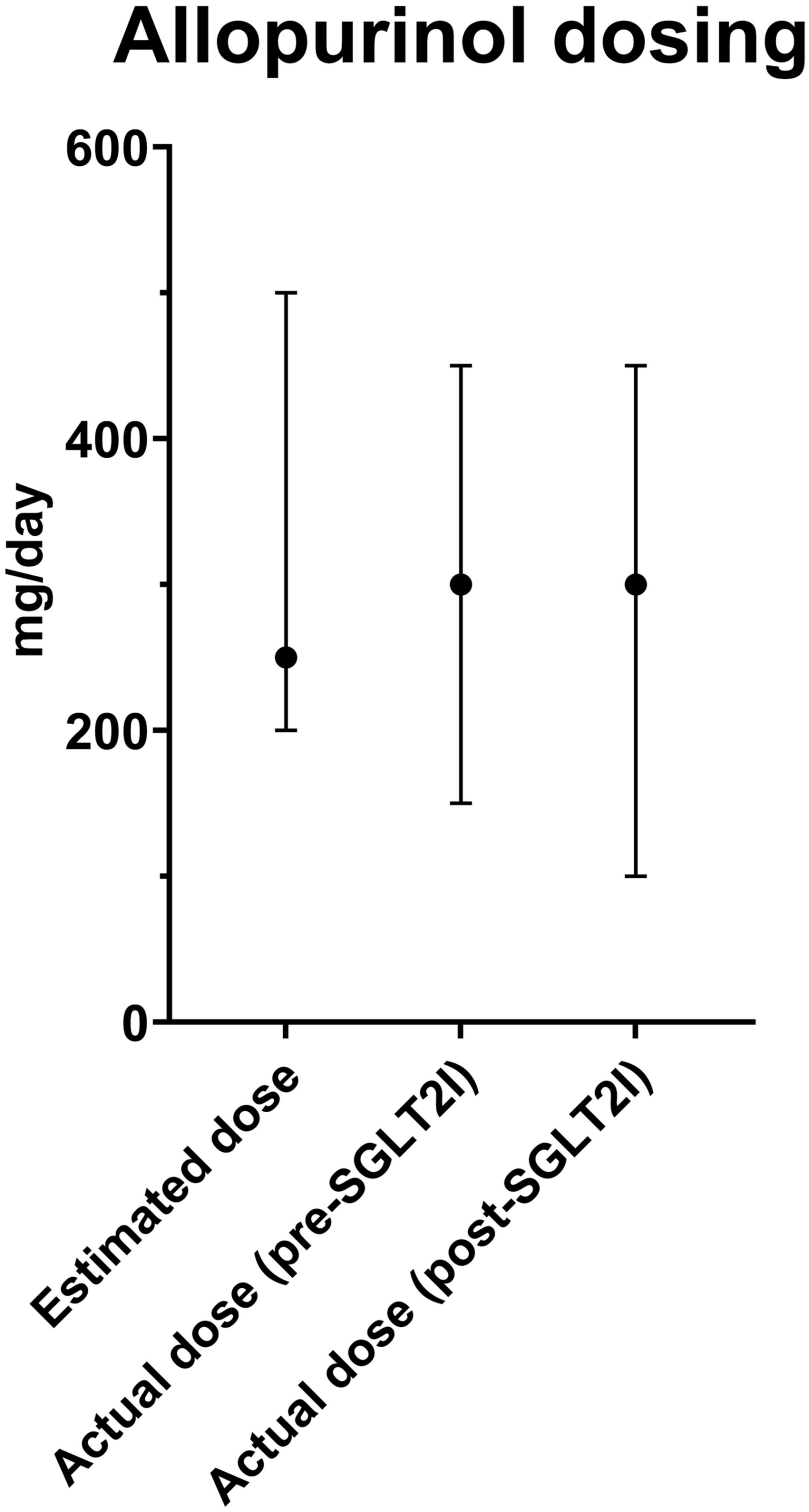
Allopurinol dosing. Median allopurinol doses (dots) with range (bars) are shown for the estimated dose, actual dose before SGLT2 inhibitor initiation (pre-SGLT2I), and actual dose after combined therapy with SGLT2 inhibitor initiation (post-SGLT2I).

No significant differences were observed in the median allopurinol dose predicted with the Easy-Allo tool (300 mg/day) and the median dose finally prescribed (300 mg/day) after initiation of combination therapy (Figure 2). However, the IQR of prescribed allopurinol doses is greater and includes lower values than the IQR of estimated doses. No significant differences were noted in patients requiring a dose higher than 300 mg/day (21.4% predicted, 20.4% prescribed under combination, *p* = 1.000).

We observed variations in several laboratory variables after initiation of combination treatment. There was a significant reduction in fasting glucose [from 121 mg/dL (IQR 155.25, 103.75) to 106 mg/dL (IQR 131.50, 89.0); *p* = 0.001], HbA1c [from 6.5% (IQR 7.33, 5.80%) to 6.1% (IQR 6.75, 5.60%); *p* = 0.007], and CRP levels [from 0.37 mg/dL (IQR 0.86, 0.21) to 0.23 mg/dL (IQR 0.38, 0.13); *p* = 0.009]. No significant changes were observed in UACR [from 21.5 mg/g (IQR 80.25, 7.75) to 19 mg/g (IQR 76.0, 7.0); *p* = 0.363] or eGFR [50.2 mL/min (IQR 79.70, 39.80) vs. 57.6 mL/min (IQR 83.72, 39.90); *p* = 0.420].

## 4 Discussion

The findings of this real-world study in 46 people with gout receiving treatment at a single specialized clinic highlight the positive impact of combining SGLT2 inhibitors with ULT (particularly XOIs). Combination treatment led to significant SU reduction, achievement of urate targets, reduction in diuretics, and likely a lower allopurinol dosage requirement. While SU reduction was greater in people without prior ULT, the attainment of SU targets was not influenced by previous ULT use. Outcomes were not influenced by patients’ comorbidities, though age and BMI might have a role in the urate response. In addition, we found improvements in several metabolic and inflammatory markers, and stable kidney function.

The high prevalence of comorbidities such as cardiovascular and renal disease among people with gout influences treatment outcomes and complicates the use of ULT (27, 30). In gout patients with high comorbidity, SGLT2 inhibitors may be of special interest. Our data suggest that comorbidities had no impact on achieving notable SU reduction and attaining SU targets with combination treatment. This finding is particularly interesting given the paucity of data from controlled trials, and because no prior study has assessed SGLT2 inhibitors in clinical practice for gout patients managed with a treat-to-crystal dissolution strategy.

In our sample, combined treatment led to a statistically significant reduction in SU levels (median of 0.85 mg/dL), slightly greater than the pooled reduction from trials in non-gout populations (18, 30). Previous studies reported SU reductions of 1.11 mg/dL with empagliflozin, 0.84 mg/dL with dapagliflozin, and 0.39 mg/dL with canagliflozin (6). We found consistent results in terms of SU reduction with dapagliflozin (1.0 mg/dL) and empagliflozin (0.9 mg/dL) but a worse result for canagliflozin (0.0 mg/dL). SU reduction was significantly more pronounced in people without prior ULT in our sample. This finding might be partly explained by the higher baseline SU level, since pharmacological interventions typically result in more substantial reductions in people who initially exhibit higher levels. In addition, SGLT2 inhibitors can complement ULT and improve treatment outcomes even in patients with better baseline performance. There is a need for randomized clinical trials to compare the SGLT2I plus ULT combination against ULT monotherapy and robustly confirm the effect of SGLT2 inhibitors on therapeutic outcomes. We acknowledge that the observed effects seem lower than those reported with the combination of XOIs and uricosurics such as benzbromarone (31). Still, the additional cardiometabolic benefits of SGLT2 inhibitors would support their use.

The attainment of therapeutic SU goals in our sample surpassed previously reported rates in rheumatology clinical practice (6), with almost all participants reaching levels below 6 mg/dL, and over three-quarters also achieving the stricter target (<5 mg/dL), which is aimed at people with more severe forms of gout. Reaching this target in a population with high comorbidity is relevant, as it shows combination treatment could lead to reduced long-term complications and an improved quality of life for these high-risk patients (32). Interestingly, target achievement was unrelated to the type of SGLT2 inhibitor (at least for empagliflozin or dapagliflozin) or XOI prescribed. Age, sex, obesity, and CKD also showed no influence on the outcome. The diuretic adjustment showed no effect on attaining SU <5 mg/dL, either, despite the known urate-increasing effects of loop diuretics and thiazides. This was probably due to most participants achieving the target, a uricosuric effect of SGLT2 inhibitors stronger than the impact of mild diuretic dose adjustments, and the study reduced sample size. Compared with baseline values under monotherapy, combination therapy resulted in at least a 30% increase in the proportion of people achieving SU targets (<6 mg/dL and <5 mg/dL), highlighting the added benefit of adding SGLT2 inhibitors to ongoing ULT. These results confirm that combining SGLT2 inhibitors with ULT enhances the urate-lowering effect, enabling patients to achieve levels favorable for dissolving MSU crystal deposits (33).

Our findings also suggest that adding SGLT2 inhibitors might reduce the required dose of allopurinol, in some cases to below the predicted dose. This trend can be interpreted as an additional benefit of SGLT2 inhibitors, enabling more effective control of SU levels with lower doses of allopurinol. Using lower allopurinol doses may be particularly beneficial for people with CKD as it reduces the risk of dose-related adverse effects in this group (34). Additionally, this could represent a cost-effective approach, reducing medication burden and improving patient outcomes. We were unable to analyze dose reduction for other ULTs such as febuxostat or benzbromarone due to the lack of dosing formulas, the lower number of participants using those agents, and their limited dose range.

As well as helping to lower SU levels, our study suggests SGLT2 inhibitors may optimize gout management by improving cardio-metabolic and inflammatory parameters and reducing the need for diuretics. In published compiled data, SGLT2 inhibitors reduced HbA1c by approximately 0.5–0.8%, improved fasting plasma glucose, and promoted weight loss through increased glycosuria (17, 18, 35). Consistent with these results, we observed a 0.4% reduction in HbA1c and an improvement in fasting plasma glucose. These effects are particularly beneficial in people with insulin resistance. Our participants did not have their weight registered in their electronic medical records. In terms of renal benefits, data from large trials and guideline updates confirm that SGLT2 inhibitors stabilize eGFR decline and lower the risk of renal endpoints such as dialysis or transplant (10, 14). Our results indicated stable renal function. In terms of inflammatory markers, people with hyperuricemia have higher CRP levels (36–38), and the elevated systemic inflammation indicated by this parameter carries serious risks (2), including increased cardiovascular morbidity and mortality. The risk is particularly pronounced in people with tophi or markedly elevated SU levels. Even those with asymptomatic hyperuricemia have subclinical inflammation, and the interplay of this factor with oxidative stress may contribute to endothelial injury and progression of comorbidities. Therefore, the reduction of CRP in people receiving SGLT2 inhibitors, as observed in our study, represents an additional benefit.

Taken together, these findings highlight the potential of SGLT2 inhibitors as a valuable strategy for gout management, particularly in people with comorbid conditions such as T2D, heart failure, or CKD. The ability of these drugs to lower SU levels while offering cardiometabolic benefits could make them a useful part of a holistic approach to this complex clinical scenario (16).

This study has some limitations, including a small sample size, which may have precluded meaningful subgroup comparisons, and baseline SU levels that were already close to the target, potentially limiting the observable impact of SGLT2 inhibitors. Additionally, uncontrolled variables—such as diet or medication adherence—may have influenced the outcomes and their interpretation, and the absence of a control group meant we were unable to compare treatments in parallel. Randomized controlled trials, such as the ongoing SAVE-care trial (39), are needed to clarify the effect of SGLT2 inhibitors in this clinical scenario, although the generalizability of the results remains limited to people with similar characteristics.

Key strengths of this study include its focus on a population with a high comorbidity burden, which is representative of typical rheumatology practice, and consistent follow-up by the same specialist in a specialist gout clinic, which ensured homogeneity in diagnosis and management [treat-to-target (40) or treat-to-crystal dissolution (32)]. Our analysis also examined differences by type of ULT or SGLT2 inhibitor and included a comprehensive evaluation of multiple clinical and laboratory parameters, reinforcing the robustness of findings reported in previous studies (20).

In conclusion, combining SGLT2 inhibitors with ULT (particularly XOIs) in people with gout seen at a specialist hospital clinic was successful in achieving SU targets. Initiating SGLT2 inhibitors reduced SU levels, with higher figures observed in participants who were not on ULT. The combined approach improved urate control regardless of patient characteristics and allowed for lower allopurinol doses in some cases. It was also associated with reduced diuretic use. These positive findings from clinical practice should prompt prospective controlled studies of SGLT2 inhibitors in people with gout.

## Data Availability

The raw data supporting the conclusions of this article will be made available by the authors, without undue reservation.
