# Supplementary material for: Combination of sodium-glucose cotransporter type 2 inhibitors and urate-lowering therapy in people with gout from a specialist clinic: a retrospective, single-center observational study
Source: Front Med (Lausanne). 2026 Jan 12;12:1726111. doi: 10.3389/fmed.2025.1726111 (PMC12832974; doi:10.3389/fmed.2025.1726111)
Supplement: Supplementary file 1 [file Data_Sheet_1.DOCX]

**Combination of sodium-glucose cotransporter type 2 inhibitors and urate-lowering therapy in people with gout from a specialist clinic: a retrospective, single-center observational study.**

**Supplementary Material**

**Table S1.** Association of covariates on achievement of SU levels below 5 mg/dL in a bivariate logistic regression model.

| Covariate | OR (95%CI) | P-value |
| --- | --- | --- |
| Age, in years | 1.07 (0.99-1.14) | 0.079 |
| Age >75years | 1.32 (0.30-5.77) | 0.709 |
| Men | 0.58 (0.09-3.65) | 0.565 |
| Arterial hypertension | 0.98 (0.10-9.91) | 0.979 |
| Dyslipidemia | 1.14 (0.19-6.75) | 0.883 |
| Type 2 diabetes mellitus | 1.52 (0.14-16.91) | 0.732 |
| Ischemic cardiopathy | 0.37 (0.08-1.72) | 0.204 |
| Heart failure | 1.89 (0.41-8.78) | 0.417 |
| Tobacco | 1.15 (0.19-6.85) | 0.881 |
| Body mass index, in kg/m^2^ | 0.86 (0.72-1.02) | 0.080 |
| Body mass index ≥30kg/m^2^ | 0.32 (0.06-1.78) | 0.193 |
| Tophaceous disease | 0.75 (0.16-3.51) | 0.715 |
| Years since first flare | 1.02 (0.95-1.08) | 0.642 |
| Baseline SU, in mg/dL | 0.87 (0.65-1.15) | 0.318 |
| Baseline fasting serum glucose, in mg/dL | 1.01 (0.99-1.03) | 0.262 |
| Baseline HbA1C, in % | 1.42 (0.61-3.32) | 0.420 |
| Baseline urine glucose, in mg/dL | 1.00 (0.99-1.00) | 0.451 |
| Baseline CRP, in mg/dL | 0.84 (0.60-1.19) | 0.329 |
| Baseline eGFR, in mL/min/1.73m^2^ | 1.01 (0.97-1.04) | 0.787 |
| Baseline UACR, in mg/g | 0.99 (0.99-1.00) | 0.735 |
| Chronic kidney disease | 0.85 (0.18-3.97) | 0.832 |
| Prior ULT | 1.89 (0.20-17.98) | 0.582 |
| Type of XOI |  |  |
| - Allopurinol | 1.00 (Ref) | - |
| - Febuxostat | 2.87 (0.31-26.84) | 0.355 |
| Type of SGLT2I |  |  |
| - Empagliflozin | 1.00 (Ref) | - |
| - Dapagliflozin | 1.09 (0.22-5.45) | 0.916 |
| - Canagliflozin | 1.09 (0.09-13.78) | 0.946 |
| Use of low-dose colchicine | 0.55 (0.10-3.06) | 0.492 |
| Diuretics use | 2.12 (0.48-9.32) | 0.322 |
| Diuretic adjustment | 1.33 (0.14-13.10) | 0.805 |
| Acetylsalicylic acid use | 1.32 (0.30-5.77) | 0.709 |

SU: serum urate; CRP: C-reactive protein; eGFR: estimated glomerular filtration rate: UACR: urine albumin-to-creatinine ratio; ULT: urate-lowering therapy; XOI: xanthine oxidase inhibitor; SGLT2I: sodium-glucose cotransporter type 2 inhibitor.
